# Transfer characteristics of subretinal visual implants: corneally recorded implant responses

**DOI:** 10.1007/s10633-016-9557-7

**Published:** 2016-08-10

**Authors:** K. Stingl, K. U. Bartz-Schmidt, A. Braun, F. Gekeler, U. Greppmaier, A. Schatz, A. Stett, T. Strasser, V. Kitiratschky, E. Zrenner

**Affiliations:** 1 Centre for Ophthalmology, University of Tübingen, Schleichstr. 12-16, 72076 Tübingen, Germany; 2 Retina Implant AG, Gerhard-Kindler-Straße 8, 72770 Reutlingen, Germany; 3 Werner Reichardt Centre for Integrative Neuroscience (CIN), University of Tübingen, Schleichstr. 12-16, 72076 Tübingen, Germany; 4 Klinikum Stuttgart - Katharinenhospital, Eye Clinic, Kriegsbergstraße 60, 70174 Stuttgart, Germany; 5 NMI Natural and Medical Sciences Institute at the University of Tübingen, Markwiesenstr. 55, 72770 Reutlingen, Germany

**Keywords:** Neuroprosthetics, Retinitis pigmentosa, Artificial vision, Hereditary retinal diseases, Subretinal visual implant, Electrophysiology

## Abstract

**Purpose:**

The subretinal Alpha IMS visual implant is a CE-approved medical device for restoration of visual functions in blind patients with end-stage outer retina degeneration. We present a method to test the function of the implant objectively in vivo using standard electroretinographic equipment and to assess the devices’ parameter range for an optimal perception.

**Methods:**

Subretinal implant Alpha IMS (Retina Implant AG, Reutlingen, Germany) consists of 1500 photodiode-amplifier-electrode units and is implanted surgically into the subretinal space in blind retinitis pigmentosa patients. The voltages that regulate the amplifiers’ sensitivity (*V*_gl_) and gain (*V*_bias_), related to the perception of contrast and brightness, respectively, are adjusted manually on a handheld power supply device. Corneally recorded implant responses (CRIR) to full-field illumination with long duration flashes in various implant settings for brightness gain (*V*_bias_) and amplifiers’ sensitivity (*V*_gl_) are measured using electroretinographic setup with a Ganzfeld bowl in a protocol of increasing stimulus luminances up to 1000 cd/m^2^.

**Results:**

CRIRs are a meaningful tool for assessing the transfer characteristic curves of the electronic implant in vivo monitoring the implants’ voltage output as a function of log luminance in a sigmoidal shape. Changing the amplifiers’ sensitivity (*V*_gl_) shifts the curve left or right along the log luminance axis. Adjustment of the gain (*V*_bias_) changes the maximal output. Contrast perception is only possible within the luminance range of the increasing slope of the function.

**Conclusions:**

The technical function of subretinal visual implants can be measured objectively using a standard electroretinographic setup. CRIRs help the patient to optimise the perception by adjusting the gain and luminance range of the device and are a useful tool for clinicians to objectively assess the function of subretinal visual implants in vivo.

**Electronic supplementary material:**

The online version of this article (doi:10.1007/s10633-016-9557-7) contains supplementary material, which is available to authorized users.

## Introduction

Several therapeutic approaches are under development for hereditary degeneration of the outer retina, including gene-therapy [1–3], electrostimulation [4], and microelectronic visual implants [5–8].

**Fig. 1:**
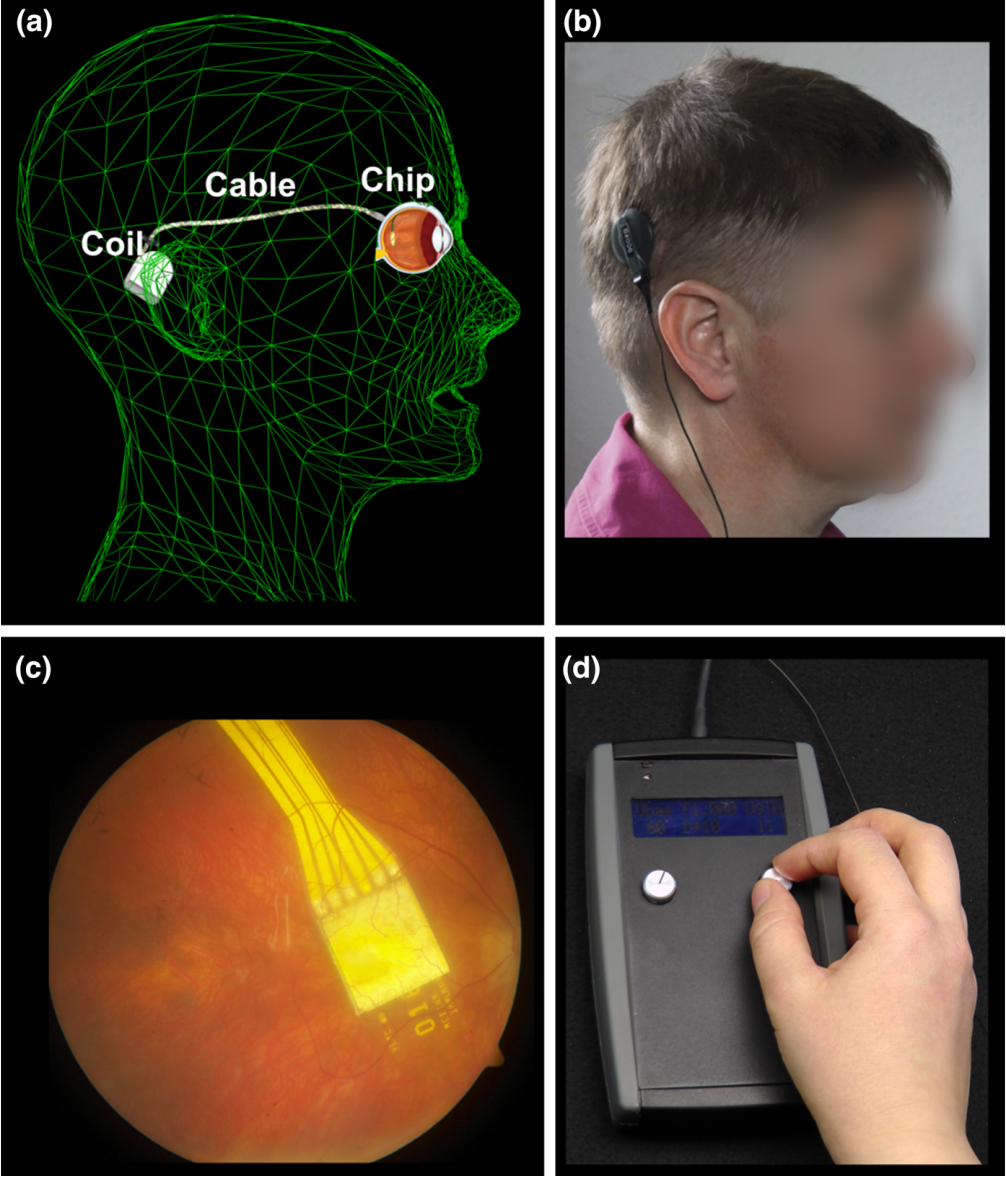
Subretinal alpha IMS implant (Retina Implant AG, Reutlingen, Germany). **a** The subdermal receiver coil behind the ear provides power and forwards control signals via a subdermal cable and a thin intraocular foil to the chip in the eye. **b** The external transmitter coil is magnetically kept in place above the subdermal coil behind the ear and provides power and signals via transdermal electric induction. **c** The chip is placed surgically beneath the fovea and contains 1500 pixels (independent microphotodiode-amplifier-electrode elements) on a 3 mm × 3 mm area. **d** Via a thin black cable, a small battery pack powers the primary external coil. Settings of contrast sensitivity and brightness can be adapted manually. (Figure modified from Stingl et al. [6]

In this paper, we present a method to assess the transfer characteristics of the subretinal visual Retina Implant Alpha IMS objectively in vivo. Retina Implant Alpha IMS (Retina Implant AG, Reutlingen, Germany, Fig. 1) is a CE-approved medical device which can partially replace lost photoreceptor function by performing the light-to-voltage conversion. This results in a current injection and a charge transfer to retinal neurons at each of its 1500 electrodes, mainly stimulating bipolar cells [12, 13]. The Alpha IMS implant can be implanted in blind patients suffering from hereditary degeneration of photoreceptors such as retinitis pigmentosa. Because in retinitis pigmentosa the inner retina and the remaining visual pathway stay largely functional even during end-stage conditions [8], spatially ordered electrical stimulation can activate the natural visual pathway from the bipolar cells onward. Patients report to perceive an electrical grey-scale image within a square-shaped 11° × 11° visual field [6, 9]. To date, the subretinal visual implant has been shown to restore useful visual functions in blind patients as well as to provide a visual acuity of up to 20/546 (decimal 0.037) and improve performance in tasks such as object localisation and recognition in daily life [5, 6, 10]. From 2010 to 2013, the implant was applied in a multicentre clinical trial in 29 patients with end-stage hereditary retinal disease (www.clinicaltrials.gov, NCT01024803) [6, 10, 11].

The active subretinal chip is surgically implanted into the subretinal space (Fig. 1c), connected via a subdermal cable to a retroauricular receiver coil (Fig. 1a) that is powered inductively via an epidermal transmitter coil controlled by an external handheld unit (Fig. 1b, d), held in place by a subdermal magnet. The active subretinal chip is a microphotodiode array (MPDA) that consists of 1500 independent microphotodiode-amplifier-electrode units. At each electrode of the MPDA, rectangular anodic voltage pulses are applied and current is injected into the retina [5], where the amplitude of the voltage pulses depends on the luminance present at the corresponding microphotodiode. The function of the corresponding charge output per pixel plotted against the logarithm of illuminance, the so-called transfer characteristic curve in vitro, is being measured in vitro after manufacturing the implant and has a sigmoidal shape. The steep part of the sigmoid covers approximately two logarithmic units and represents the range where different illuminations are translated into a graded electrical output of different magnitude, building a basis for contrast perception.

In contrast to natural vision, there is no luminance adaptation of the photodiode system. Instead, the transfer characteristic curve can be shifted across the luminance spectrum by adjusting the control voltage (*V*_gl_), allowing an adjustment of contrast perception to ambient light levels. Additionally, the amplifier gain can be adjusted using a second control voltage (*V*_bias_), changing the slope and the maximal voltage output, (brightness) of the perception. The transfer characteristic curves are measured in vitro for various combinations of the *V*_gl_ and *V*_bias_ parameters. A patient with the Retina Implant Alpha IMS can manually adjust both parameters on the handheld unit (Fig. 1d) in order to optimise his/her visual perception. This optimisation requires some training supported by a specialised ophthalmologist, clinical engineer, or mobility trainer during the first few days after implant activation.

Here, we describe a new method to test the transfer characteristic curve of the subretinal visual implant Alpha IMS objectively in vivo using standard electroretinographic equipment with long duration light flashes.

Generated by the voltage pulses of the chip, current spreads from the electrodes through the retina and tissue back to the return electrode and at each point along the current path a voltage drop occurs. Consequently, at the cornea, voltage pulses can be recorded with an amplitude that correlates with the amplitude of the chip output voltage, which have a time course that is congruent to the time course of the chip-induced current pulses. We measured these corneally recorded implant responses (CRIR) to full-field stimulation with standard electroretinographic equipment picking up the voltage at the cornea by a corneal electrode. This method presents (1) an important measurement to determine the optimal settings according to luminance conditions and (2) an objective diagnostic tool for measuring the implant function in vivo.

## Materials and methods

### Alpha IMS subretinal visual implant

The subretinal Alpha IMS implant (Fig. 1) is a CE-approved medical device developed for the restoration of visual functions in blind patients suffering from photoreceptor degeneration. A 1500-pixel chip forms the implant’s core. Each pixel is composed of a photodiode receiving the incoming light, a differential amplification circuit to provide adequate stimulation current, and a metal electrode of 50 µm × 50 µm to transfer charges to the adjacent retinal layers. The chip is approximately 3 mm × 3 mm in size and 70 µm thick, mounted on polyimide foil (thickness approx. 20 µm) and encapsulated with an electrically isolating biocompatible layer [5, 6]. The polyimide foil exits the subretinal space in the upper temporal periphery through the choroid and the sclera, where it is connected to the power supply cable, leading to the retroauricularly placed subdermal receiver coil (Fig. 1a, c). Here, an external transmitter coil placed on top of the skin behind the ear (Fig. 1b), held in place by a magnet permits an inductive transfer of energy and control signals from the handheld unit (Fig. 1d) through the skin to the implant.

The external handheld unit (Fig. 1d) has two knobs. One knob changes the gain of the amplifiers, i.e., it compresses or stretches the transfer characteristic function to a lower or higher maximum output by changing the *V*_bias_ voltage, thereby changing the maximal brightness perception. The other knob varies the absolute sensitivity (the *V*_gl_ voltage), i.e., it shifts the transfer function along the range of luminances. Both knobs have arbitrary values from 0 to 99 indicated on a small display. The range of voltage provided within these 100 units is adjusted individually for each patient in the present version of the power supply.

The chip typically records and transfers images 5 times per second (a working frequency of 5 Hz, adjustable from 1 to 20 Hz) and provides a “point-by-point electrical image” of the luminance distribution. The output signal of each electrode of the implant is a monophasic anodic voltage pulse of 1 ms (adjustable from 0.1 to 2 ms). Due to the capacitive properties of the interface between electrode and retina, the voltage pulse gives rise to a biphasic current pulse. The current spreads through the retina and depolarises the bipolar cells [12, 13]. From the bipolar cells onward, the signal is processed via the remaining visual pathway. The perceived image is a flickering image in grey levels within a squared field spanning 15° across corners [6, 10].

### Patient characteristics

Patients who receive the subretinal visual implants suffer from end-stage hereditary degeneration of the outer retina, e.g., retinitis pigmentosa. Between 2010 and 2013, 29 patients worldwide received the Retina Implant Alpha IMS in their least functional eye in a multicentre clinical trial (www.clinicaltrials.gov, NCT01024803) [9]. Any additional diseases of the eye that affect the visual pathway or clear optical media are contraindications for a subretinal visual implant (e.g., glaucoma, diabetic retinopathy, occipital stroke, profound amblyopia, congenital blindness, corneal opacities). Written informed consent in accordance with Declaration of Helsinki was obtained from all participants. The study was approved by the local ethics committee.  

### Recording protocol

**Fig. 2:**
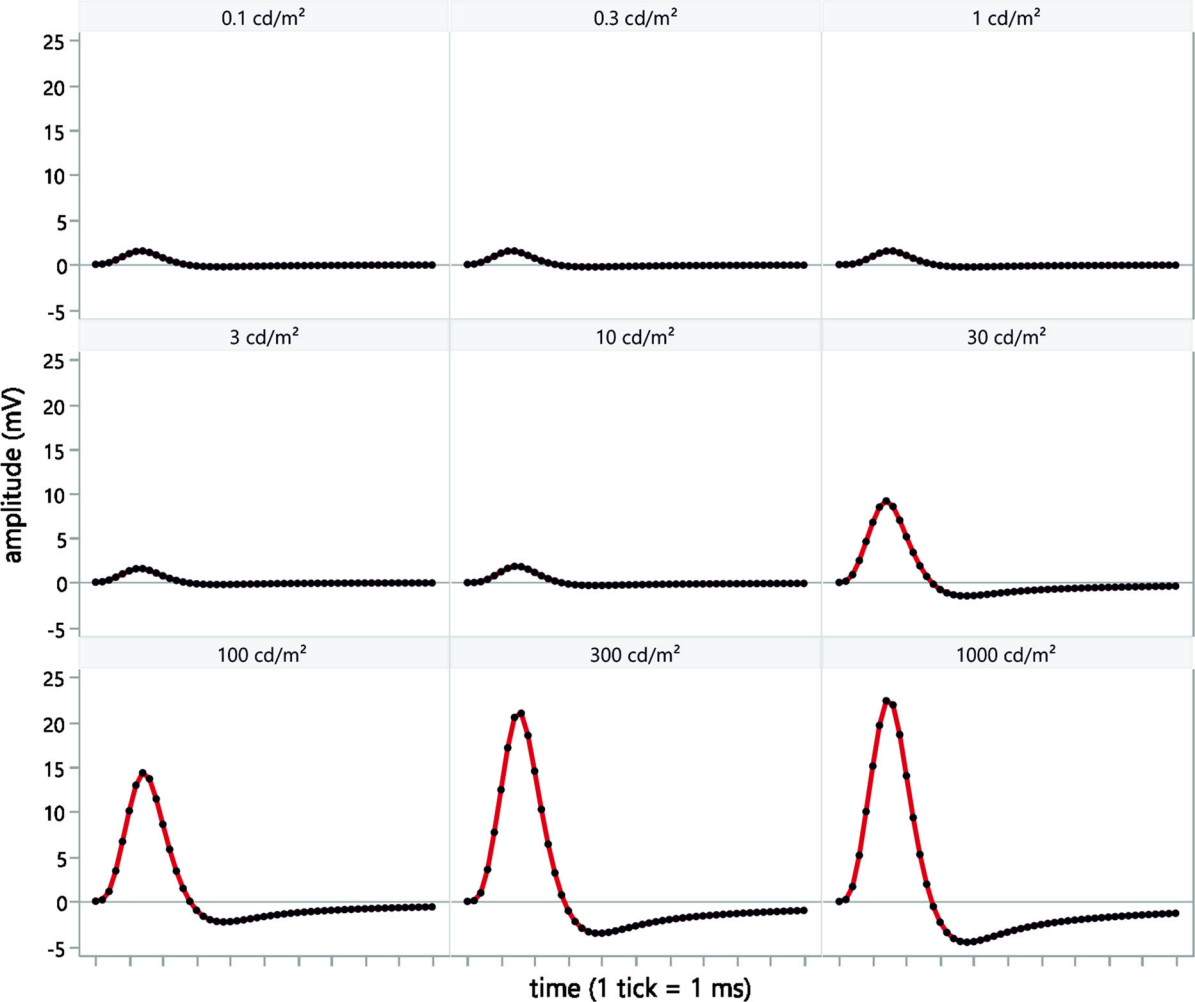
Examples of in vivo CRIR recordings with 9 luminance steps showing a typical pulse shape with the sampling frequency of 5 kHz (*black dots*). The 1 ms anodic voltage pulse output from the implant electrodes results in a slowly rising current that shows an overshoot in the opposite direction after pulse offset and a subsequent return to the baseline. The full-field luminance levels for each particular step are indicated above each curve. With increasing luminance, the amplitude of the CRIR increases showing the dynamic range of the particular chip settings (here *V*_gl_ = 20, *V*_bias_ = 65). These amplitudes are then documented as a CRIR curve (as shown in Fig. 3)

CRIRs (see Fig. 2) were obtained using a ColorDome^®^ controlled by an Espion E^2®^ module (Diagnosys LLC, Cambridge, UK). The pupil was dilated to guarantee the highest possible illumination of the MPDA surface. The partner eye was occluded, and its responses were not recorded. DTL electrodes [14, 15] (conjunctival, active electrode) and two gold-cup electrodes (VIASYS Healthcare, Warwick, UK) were positioned in the same way as for full-field electroretinography: with one on the forehead (ground electrode) and a second temporal to the lateral canthus of the study eye (reference electrode). Skin-cleaning procedures with an abrasive gel were performed before electrode positioning to achieve an acceptable impedance level (≤5 kΩ). Any corneal electrode can be used; however, postoperatively the DTL is the least invasive.

Dark adaptation was not necessary (as no adaptation mechanisms are present in the MPDA), but the room was completely darkened during the measurements to avoid contributions from room illumination to CRIR. The MPDA is sensitive to infrared light. Therefore, the ColorDome^®^ internal camera, which uses infrared light, was switched off for all measurements.

The ERG protocol consisted of 9 steps of long duration flashes (350 ms), with increasing stimulus luminance of white light from 0.1 to 1000 photopic cd/m^2^ in 0.5 log steps using white mixed light (LED spectrum: blue 470 nm, amber 594 nm, green 513 nm, red 635 nm). The photodiodes cover the visible light spectrum and partially infrared irradiation (sensitivity range approx. 400–950 nm). Each 9-step procedure was performed for a specified contrast (*V*_gl_) and gain (*V*_bias_) combination setting of the implant. The working frequency (5 Hz) and pulse duration of the chip (1 ms) were kept constant for the entire measurement, so that for each step, one or two chip responses were recorded. All details of the protocol settings are described in Supplementary Table 1.

The Espion E^2^ module uses a DC amplifier with an input range of the ±0.5 V (CMRR > 100 dB at 50 Hz) and a fixed gain of 10. Signals are digitised using a 16 bit ADC with 12 bit DC offset (internal bias, which creates an offset voltage, stored as a 12 bit value). The recordings were observed continuously. A saturation of the recorded signal was not observed (would present as a ceiling flatline in the shape of the signal).

For recording the CRIR, a sampling frequency of 5 kHz was used and signals were filtered using a build-in digital band-pass filter (second order cascaded Bessel filter) with a low cut-off frequency of 0.312 Hz and high cut-off frequency of 300 Hz. During the examination, recorded signals are observed continuously for potential artefacts. In case of an artefact, the particular luminance step is repeated.

The amplitudes were read off the screen and manually documented in an Excel worksheet to create CRIR values (see Results).

## Results

A typical pulse shape of the voltage at the cornea generated by a 1 ms anodic voltage pulse of the chip and recorded with the ERG equipment is shown in Fig. 2. This CRIR is the typical pulse response of the amplifier and Bessel filter to the biphasic voltage pulse on the cornea.

**Fig. 3:**
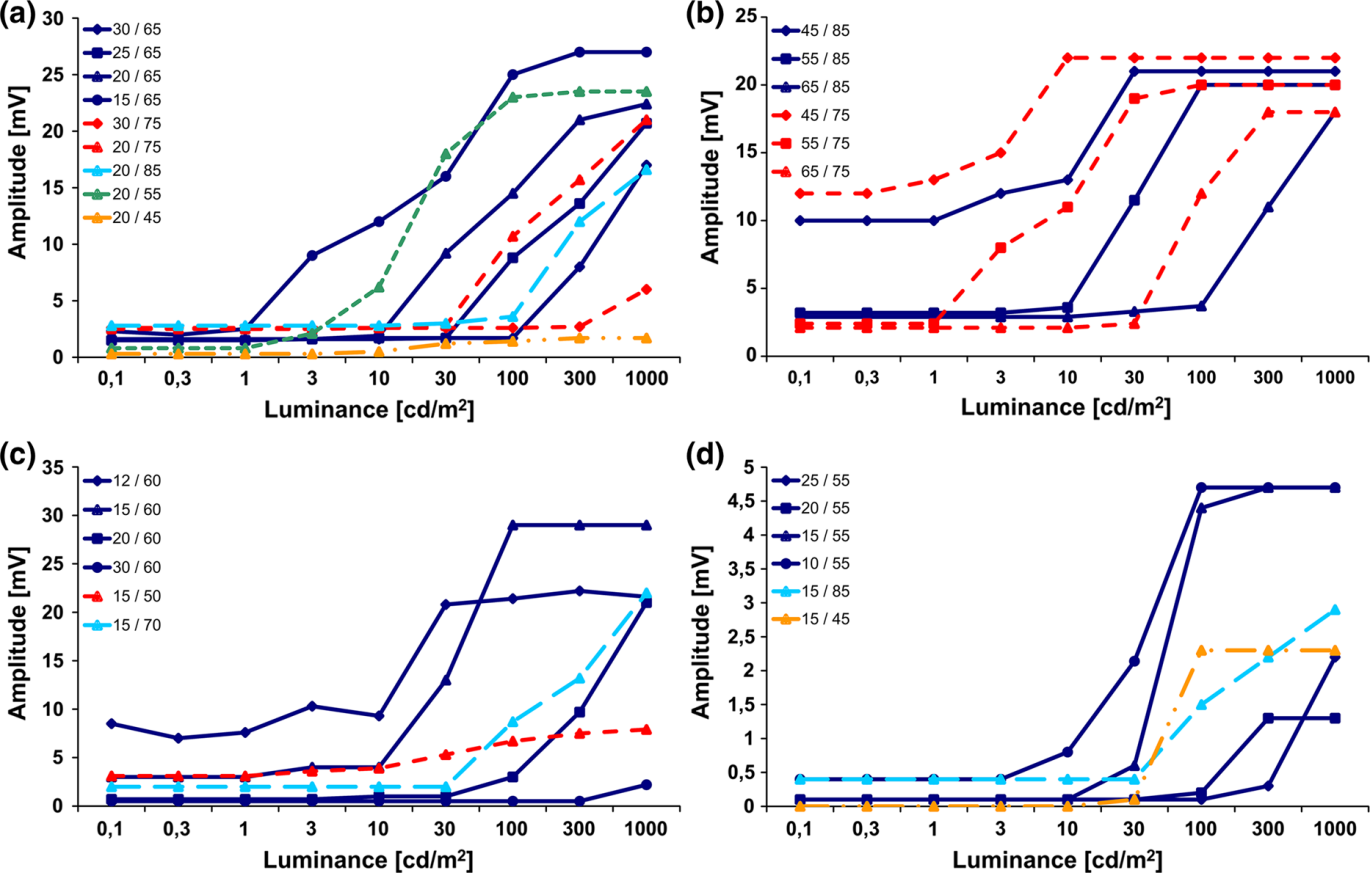
CRIR as transfer characteristic curves in vivo in various combinations of the *V*_gl_/*V*_bias_ settings that define the MPDA-amplifiers’ sensitivity and gain, respectively, from four patients measured at various time points during the 1-year follow-up visits. **a** patient C9, **b** patient C10, **c** patient C12 **d** patient C7. The inserted tables show values for *V*_gl_/*V*_bias_

**Fig. 4:**
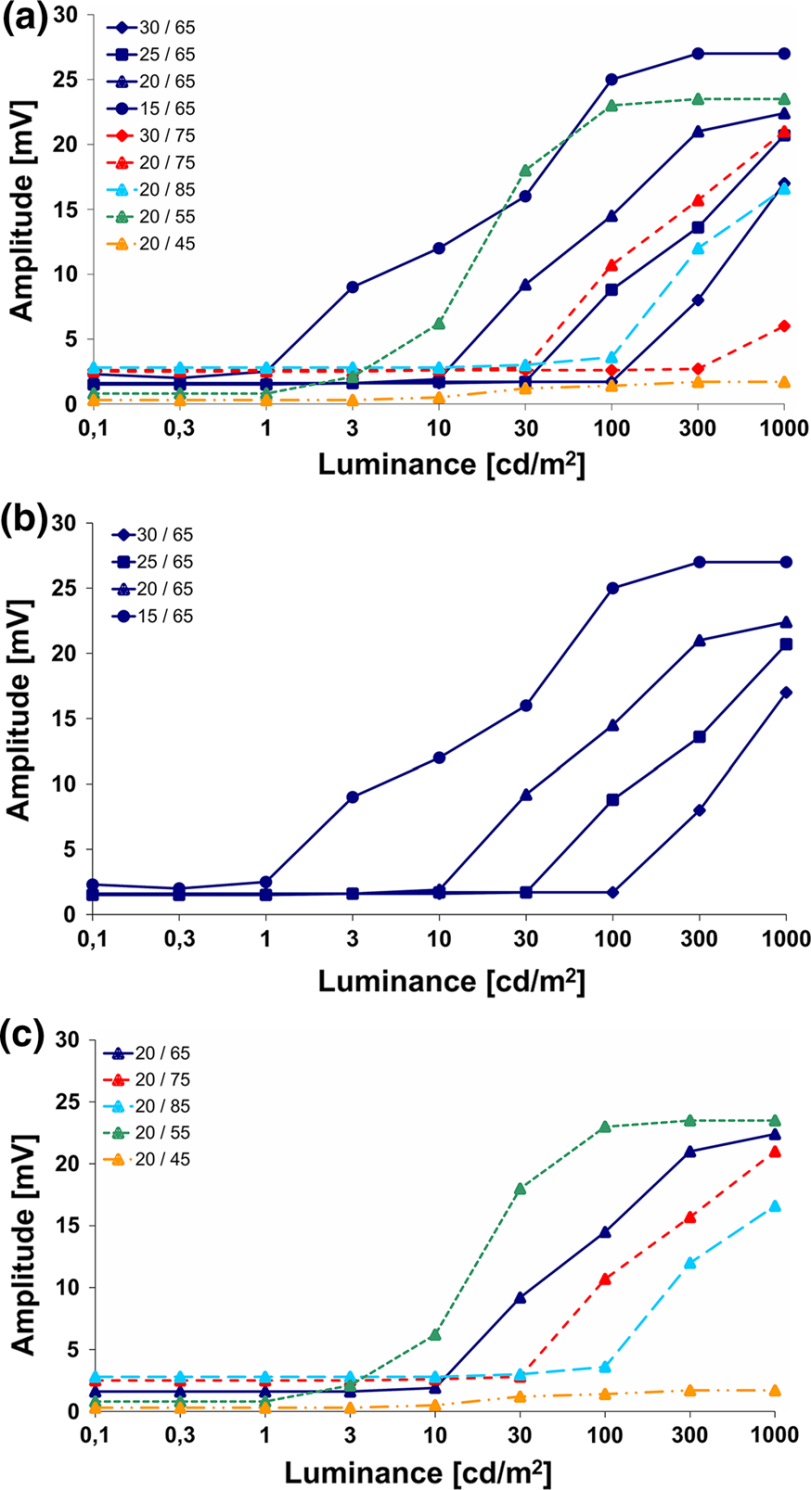
**a** CRIR from patient C9, as taken from Fig. 4a, shows data measured during a single trial visit; **b** illustrates how changing the *V*_gl_ value shifts the curve along the luminance range; **c** illustrates the change of minimum and maximum output when altering *V*_bias_. The values for *V*_gl_/*V*_bias_ are shown in the *upper left corner*

A series of CRIRs *(*Fig. 2) evoked by increasing steps of stimulus luminance allows to determine the transfer characteristic curve of the Retina Implant Alpha IMS in vivo, resulting in a sigmoid curve describing the corneally recorded voltage as a function of log luminance (Figs. 3, 4). In Fig. 3, examples of four patients are shown as separate diagrams. A single curve in each of the diagrams represents a CRIR transfer function recorded with specific *V*_gl_/*V*_bias_ combination. The respective *V*_gl_/*V*_bias_ values of the colour-coded curves are shown in the left upper part of the figure. The CRIR measurement in each patient consists of several curves based on different *V*_gl_/*V*_bias_ combinations.

As the amplifier in the MPDA covers only a range of approximately two logarithmic units of luminance, its sensitivity has to be adjusted to cover the range prevailing ambient luminance. Turning the sensitivity knob of the handheld control box (change in *V*_gl_ 0–99) shifts the CRIR curve left or right (Fig. 4b). Variation of the amplifiers’ gain (*V*_bias_ 0–99) changes the maximal output of the chip, thus changing the slope and the saturation level (Fig. 4c).

Luminance steps either within the subthreshold region or saturation region of the CRIR function cannot be resolved by the implant and are therefore not seen by the patient; only if the luminance interval is within the steep slope of the sigmoidal curve contrast border recognition is possible.

Although the maximal output in the saturation region of each curve tends to be proportional to the *V*_bias_ value in vitro, a *V*_bias_ value between 55 and 75 typically produces a maximal output voltage in vivo; other *V*_bias_ values (both higher and lower) reduce the maximal outputs (Fig. 4c).

An example of settings, based on CRIRs measured in vivo for a visual test of grey level differentiation, is given in supplementary Fig. 1.

## Discussion

CRIRs are a tool for testing electronic subretinal visual implants in vivo. By recording a series of CRIRs in response to increasing full-field luminance (Fig. 2), the input/output function (Fig. 3) of the implant can be assessed. As the amplitude of the corneally recorded voltage correlates with the amplitude of the MPDA output voltage, the in vivo transfer function obtained from the CRIR amplitudes resembles the in vitro transfer function. The CRIRs cannot measure the voltage output of the device directly, but can evaluate the influence of the luminance, *V*_bias_ and *V*_gl_ variation on the measured voltage, thus monitoring the main technical function of the implant—the transfer of illuminance on the MPDA surface into voltage and current, respectively, according to the transfer function.

**Fig. 5:**
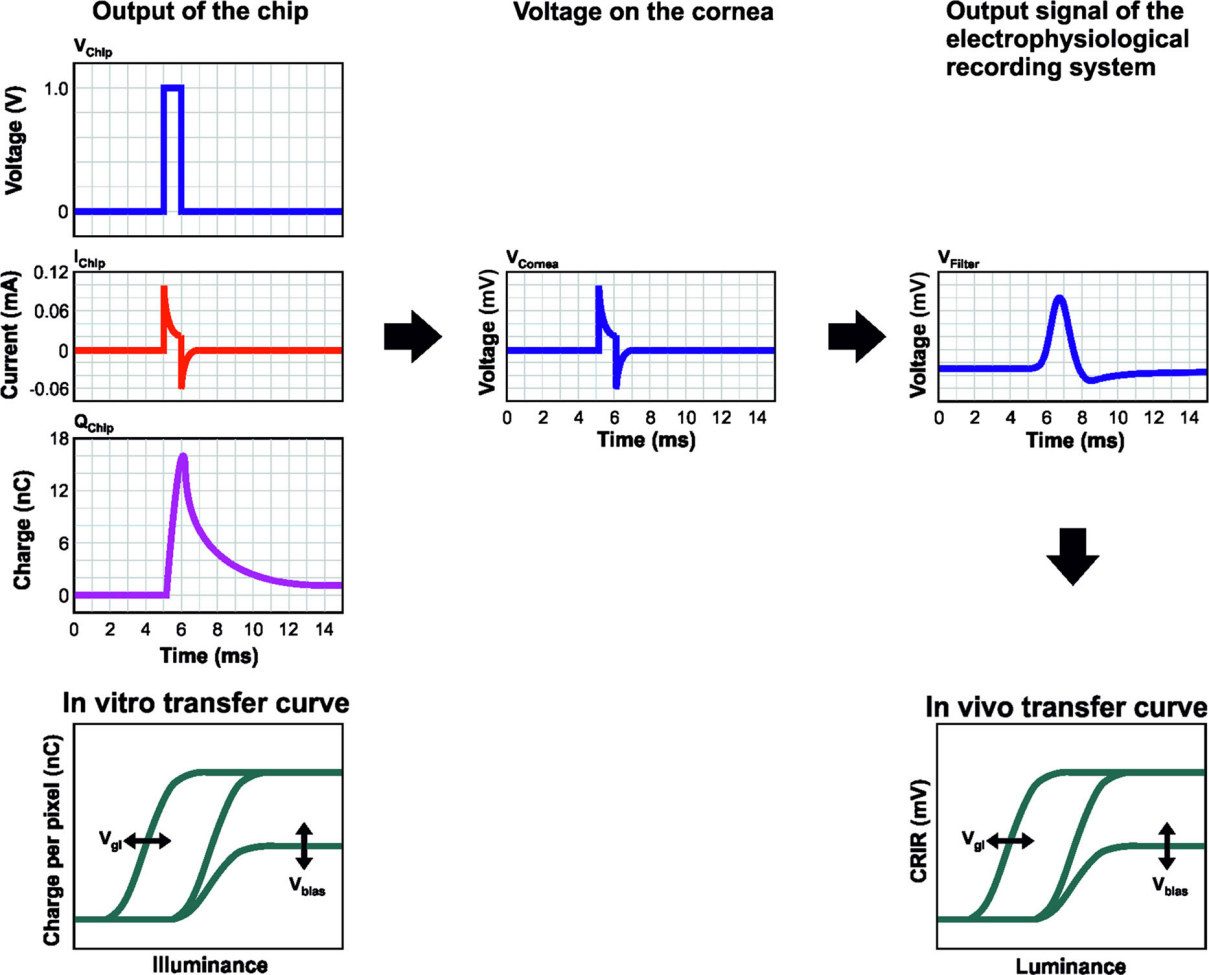
Appearance, recording and characteristics of CRIRs. *Left* Output voltage (*V*_chip_), current (*I*_chip_) and charge per pulse (*Q*_chip_) of the MPDA. From the peak charge, the in vitro transfer curve as a function of illuminance is obtained. *Middle* Related to the current spread through the tissue, a voltage pulse at the cornea (*V*_cornea_) is observable. *Right* Voltage signal (*V*_Filter_) recorded with the ERG system. From the peak value of this filtered signal (CRIR), the in vivo transfer curve as a function of luminance is obtained. Details see text

Amplitude and shape of the CRIRs depend on many factors and do not correlate solely with the actual voltage output of the device. The capacity of the electrodes on the MPDA, the contact surface between the electrodes and the retinal tissue, the resistance of the tissue itself and the impedance of the ERG electrode influences the CRIRs. Of great importance is the characteristic of the Bessel filter of ERG equipment. Mathematically spoken, the signal output from the ERG system is the convolution of the impulse response of the Bessel filter with the voltage pulse on the cornea. This gives rise for a signal delay and a pulse broadening. Thus, as the recorded signal is a surrogate marker of many factors, the shape of the corneally recorded waveform cannot be used for a detailed analysis of the MPDA and its functional integration into the retina (Fig. 5). To examine the contact between the MPDA and the retina, high-resolution OCT can be used. Especially fluid between the retina and the microchip or detachment from the chip by subretinal debris after the surgery or by pigment clumps is visible in OCT.

However, the amplitude of the signal output of ERG system is linearly dependent on the amplitude of the MPDA output. Thus, the CRIRs are well suited to verify the transfer function of the MPDA in vivo. These curves allow advising the patient during the learning phase in order to help him or her to more easily find optimal settings of brightness and contrast, preparing the patient for optimal adjustment of the settings for daily use. Before measuring visual functions on a monitor screen or in daily life, the examiner chooses a particular input/output function of the implant by selecting a *V*_gl_/*V*_bias_ combination that allows the patient to optimally distinguish luminance contrasts in a particular environment. This procedure, based on recorded CRIRs, compensates for the absent automatic adaptation to ambient luminance conditions in artificial vision, in contrast to natural vision.

Furthermore, it is important for objectively measuring the visual implants’ function.

CRIRs are recorded immediately after the implant is turned on for the first time to help to optimally set the brightness and contrast parameters in a given ambient luminance. Over time, the patient learns to change the parameters according to the subjective perception of brightness and contrast in order to achieve optimal visual perception, and subsequently CRIR is only required to control implant function over time.

CRIRs are measured over 4–5 logarithmic units of luminance. A maximum of luminance of 1000 cd/m^2^ is given due to the limitation of the electroretinographical setup; however, in reality, higher luminance conditions can be processed by the MPDA. A CRIR curve with a steep slope allows for a better differentiation between small luminance steps on the expense of the resolvable luminance range. A flatter slope would allow a larger luminance range on the expense of discernible luminances (e.g., CRIR curve in Supplementary Fig. 1). The subthreshold region of the curve indicates a luminance range where no perception is possible due to low voltage output. The saturation region indicates a luminance region in which very bright lights of different luminance are no longer differentiated. Differentiation of luminances is possible only in the slope region of the curve as different levels of grey. Patients carrying the subretinal implant describe their artificial vision as blurred images slightly flickering in various grey scales, similar to an image from an older black-and-white television [6, 10], if the implant parameters are correctly adjusted to the luminance conditions.

Recorded CRIR signals do not by themselves indicate a subjective perception. Our experiences show that the “lowest necessary” amplitude for a perception vary inter- and intra individually between the tests although recorded in many instances. This link between CRIR amplitude and subjective perception threshold has not been assessed systematically during the recordings and is thus not presented in this manuscript.

On the other hand, the fact that the objectively recorded CRIR signal and the subjective perception are not necessarily correlated each time means that CRIRs are important for objectively testing implant function. In the case of a subjective change or sudden perception failure, the voltage output can be tested objectively to distinguish biological from technical problems. For example, following a sudden loss of perception due to a damaged wire, no CRIRs are measurable. If the conducting wires have a suspected loose connection, a simple examination involving recording in all gaze directions can be very helpful; a non-regular signal can typically be measured in some eye positions with a loose cable connection. This case can be distinguished from a biological problem or retinal damage, which does not allow neuronal processing of the normally recordable chip signal, e.g., in cases where retinal blood perfusion is disturbed. In such situations, degeneration has affected the inner retina to such a degree where electrical signals can no longer excite neurons in a spatially ordered manner.

There are typically several *V*_gl_/*V*_bias_ combinations for which the slope covers a given luminance and voltage range for each patient (Fig. 3). For safety reasons, one should always aim for a curve that allows for good perception with the lowest possible voltage output. A curve in which the subthreshold region for low luminance levels has the minimal output can be considered optimal because dimmer light levels produce no sufficient charge transfer; bipolar cells [12] are thus not unnecessarily stimulated in the darker range. Similarly, if several curves that differ in their maximum outputs in the saturation region lead to a comparable perception of brightness, the output with the lowest saturation voltage should be used. CRIR curves with elevated voltages in the low luminance part (an example can be found in Fig. 3b, curves 45/85 or 45/75) are undesirable as such settings provide continuous stimulation also in the dark and reduce the contrast range.

Standard equipment for clinical ERG recording is suitable for recordings of CRIR; however, following parameters must be met:

1. Sampling frequency of 5 kHz (at least 1 kHz) with implant signal duration of 1 ms to allow for sufficient data per implant response.
2. The amplitude of the response can reach up to 35 mV. The range of the input stage of the amplifier needs to be adjusted to this signal size to prevent saturation of the amplitude or the analogue–digital converter in the ERG recording device. If the device is not adjustable, a simple alternator can be put between the electrode and amplifier input. Usually commercial clinical electrophysiological devices are built to handle larger signals.
3. The luminance of the stimulator should reach at least 500 cd/m^2^ in order to allow for testing the implant function in typical light levels of activities of daily living.

## Conclusions

The electric function of subretinal visual microelectronic implants can be measured objectively after implantation using special protocols and standard electroretinographic equipment. The assessment of corneally recorded implant responses (CRIRs) is necessary to define the optimal parameter settings for devices such as the subretinal Retina Implant Alpha IMS, particularly during the training period in order to achieve optimal contrast vision, and to objectively measure implant functions in vivo. Suboptimal results in contrast perception and saturation can be avoided by assessing CRIRs and using appropriate settings of stimulation currents.

## Electronic supplementary material

Below is the link to the electronic supplementary material.

Supplementary material 1 (DOC 54 kb)

Supplementary Fig. 1 A patient’s CRIR applied while he performs a visual task of distinguishing grey levels. The implant setting was 55/10 [*V*_bias_ / *V*_gl_]. In the task, one of six levels of grey are presented together with an adjacent area of intermediate grey level . The patient has to indicate which of the presented two grey levels is the brighter one. The patient shown here correctly distinguished 5 of 6 pairs of randomly presented grey levels lying along the slope of the CRIR curve. The intermediate grey scale (labelled with “C”) served as the comparison for all other grey scales. The number above the bar indicates the Michelson contrast. * indicates correct recognition in the test (TIFF 1292 kb)
