# Supplementary material for: Transfer characteristics of subretinal visual implants: corneally recorded implant responses
Source: Doc Ophthalmol. 2016 Aug 10;133(2):81–90. doi: 10.1007/s10633-016-9557-7 (PMC5052310; doi:10.1007/s10633-016-9557-7)
Supplement: Supplementary file 1 — Supplementary material 1 (DOC 54 kb) [file 10633_2016_9557_MOESM1_ESM.doc]

| **Global** | **Step 1** | **Step 2** | **Step 3** | **Step 4** | … | **Step 9** |
| --- | --- | --- | --- | --- | --- | --- |
| Description [cd/m^2^] | -1.0 log | -0.5 log | 0 log | 0.5 log | … | 3 log |
| Enable camera  Averages per step  Results per run | Off  1  1 | Off  1  1 | Off  1  1 | Off  1  1 | …  …  … | Off  1  1 |
| **Acquisition** | | | | | | |
| Sample frequency | 5000 Hz | 5000 Hz | 5000 Hz | 5000 Hz | … | 5000 Hz |
| Sweep pre-trigger time | 20 ms | 20 ms | 20 ms | 20 ms | … | 20 ms |
| Sweep post-trigger time | 400 ms | 400 ms | 400 ms | 400 ms | … | 400 ms |
| Sweeps per average | 1 | 1 | 1 | 1 | … | 1 |
| Interstimulus interval | 0 ms | 0 ms | 0 ms | 0 ms | … | 0 ms |
| Drift removal | Off | Off | Off | Off | … | Off |
| Manual rejection of sweeps | On | On | On | On | … | On |
| DC off-set removal  Auto-zero  Auto-zero pre-trigger | On:  0 ms  ON  20 ms | On:  0 ms  ON  20 ms | On:  0 ms  ON  20 ms | On:  0 ms  ON  20 ms | …  ...  … | On:  0 ms  ON  20 ms |
| **Stimulus** | | | | | | |
| Flash cycle | single | single | single | single | … | single |
| Flash mode  Pulse duration | pulse  350 ms | pulse  350 ms | pulse  350 ms | pulse  350 ms | …  … | pulse  350 ms |
| Stimulus luminance [phot cd/ m²] | 0.1 | 0.3 | 1 | 3 | … | 1000 |
| Stimulus colour | White 6500 K | White 6500 K | White 6500 K | White 6500 K | … | White 6500 K |
| Background luminance | 0 cd/m^2^ | 0 cd/m^2^ | 0 cd/m^2^ | 0 cd/m^2^ | … | 0 cd/m^2^ |
| **Channel 1** | | | | | | |
| Filter low-frequency cutoff | 0.3 Hz | 0.3 Hz | 0.3 Hz | 0.3 Hz | … | 0.3 Hz |
| Filter high-frequency cutoff | 300 Hz | 300 Hz | 300 Hz | 300 Hz | … | 300 Hz |
| Y-axis scaling | 20 mV | 20 mV | 20 mV | 20 mV | … | 20 mV |

Supplement Table 1: Details of the protocol parameters as can be specified in the Espion E²® electroretinography system.
