# Supplementary figures and images for: Transfer characteristics of subretinal visual implants: corneally recorded implant responses
Source: Doc Ophthalmol. 2016 Aug 10;133(2):81–90. doi: 10.1007/s10633-016-9557-7 (PMC5052310; doi:10.1007/s10633-016-9557-7)

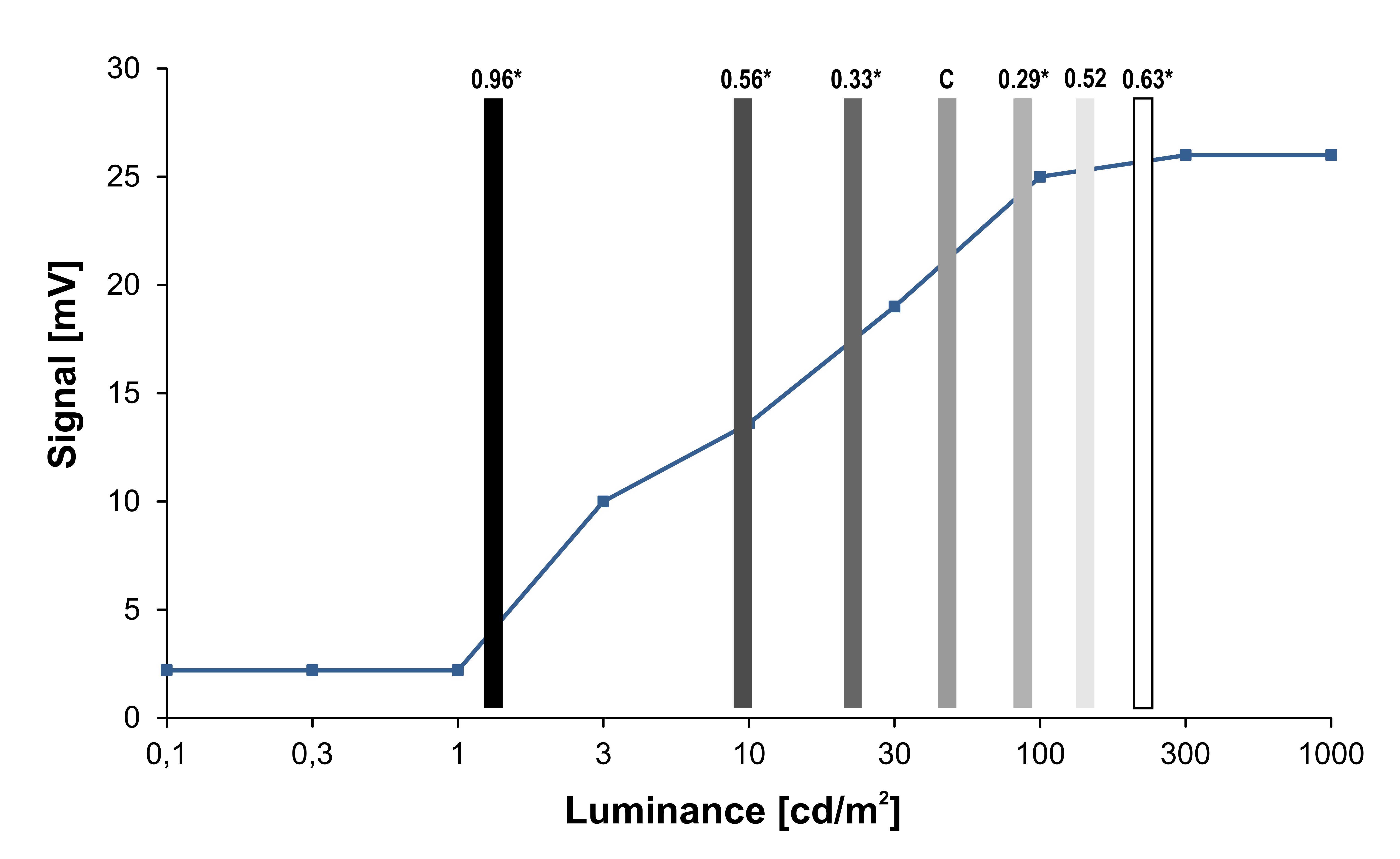

Supplement: Supplementary file 2 — A patient’s CRIR applied while he performs a visual task of distinguishing grey levels. The implant setting was 55/10 [V bias / V gl]. In the task, one of six levels of grey are presented together with an adjacent area of intermediate grey level . The patient has to indicate which of the presented two grey levels is the brighter one. The patient shown here correctly distinguished 5 of 6 pairs of randomly presented grey levels lying along the slope of the CRIR curve. The intermediate grey scale (labelled with “C”) served as the comparison for all other grey scales. The number above the bar indicates the Michelson contrast. * indicates correct recognition in the test (TIFF 1292 kb) [file 10633_2016_9557_MOESM2_ESM.tiff]
